# Clinical Significance of IgG Avidity Testing and Other Considerations in the Diagnosis of Congenital Cytomegalovirus Infection: A Review Update

**DOI:** 10.3390/medsci4010005

**Published:** 2016-03-08

**Authors:** Idris Abdullahi Nasir, Adamu Babayo, Muhammad Sagir Shehu

**Affiliations:** 1 Department of Medical Laboratory Services, University of Abuja Teaching Hospital, Gwagwalada, FCT Abuja PMB 228, Nigeria; 2 Department of Medical Microbiology and Parasitology, College of Health Sciences, University of Ilorin, PMB 1515 Ilorin, Nigeria; 3 Department of Medical Microbiology, Abubakar Tafawa Balewa University Teaching Hospital, Bauchi PMB 0117, Nigeria; Adamsbb@yahoo.com; 4 Immunology unit, Department of Medicine, Ahmadu Bello University, PMB 05 Zaria, Kaduna State, Nigeria; msgiade05@yahoo.com

**Keywords:** congenital transmission, IgG avidity, antenatal screening, cytomegalovirus

## Abstract

Prompt and accurate laboratory testing of women before or during antenatal days is necessary for detecting humoral immunological responses against cytomegalovirus (CMV) infection and assessing risk of congenital transmission. CMV is the most common viral etiology with the greatest propensity to induce neonatal pathologies. Most healthcare facilities in developing countries rely solely on anti-CMV IgM and IgG assays in diagnosing CMV infections. However, these parameters have some worrisome limitations. This study reviewed the significance of IgG avidity testing as a highly sensitive and specific tool that improves decisions regarding diagnosis of maternal and congenital CMV infections. We conducted this review from relevant published articles using an extensive literature search made through PubMed, Scopus and Google scholar on the concepts of congenital CMV (CCMV) transmission and clinical significance of IgG avidity testing in diagnosis of CCMV infections. Findings from our review revealed that IgG avidity testing in some developed societies was frequently utilized to resolve dilemmas associated with serodiagnosis of CMV infections, however, there is paucity of information in regards to its use in developing countries. The non-inclusion of IgG avidity testing during serological investigations of CMV could be a reason why congenital CMV infections and associated pathologies often go underdiagnosed in developing countries.

## 1. Introduction

Human cytomegalovirus (CMV) is a member of the herpesviridae family. It is a double stranded, enveloped and ubiquitous virus with DNA genome. Most CMV infections are beneath the threshold of clinical recognition. However, they can cause serious diseases in fetuses and in immunocompromised individuals but rarely cause disease in healthy persons [1]. Fetal CMV infection is of great public health concern because it has been established as the most common source of congenital viral infection. It has a global prevalence range between 0.2% to 2.4% of all live births [2,3]. CMV is now the most common viral etiology of mental retardation and auditory disorder of children in developed countries [4] however, such data has not been established in most developing nations. CMV causes deformation of preformed tissues rather than malformation of developing organs [4], so it can affect fetuses at any gestation age, although earlier primary infection is usually more severe [5].

Primary CMV infection is defined when a previously IgG seronegative (susceptible) person becomes infected and becomes IgM seropositive while secondary or non-primary infection is defined as intermittent detection of anti-CMV IgM antibodies (or CMV-DNA) and excretion of the virus even when the host had previous humoral immunity (*i.e.*, positive immunoglobulin G) and may be due to either reactivation of an endogenous virus [4,5] or exposure to a new CMV train from an external source [4]. Primary maternal infection is most likely to cause severe symptomatic congenital infection [4,5] but recurrent or reactivated infections may also cause significant disability. Perinatal CMV infection is usually not associated with clinical symptoms in term babies, but may also cause serious problems in preterm infants [4,5].

Despite the complexities and challenges associated with diagnosis of congenital CMV infection, important landmarks have been achieved in recent years, these included tests to determine the avidity index of anti-CMV IgG, allowing differential diagnosis of primary from non-primary CMV infections and innovative molecular techniques to detect the CMV in amniotic fluid. In this review, we presented the concepts of mother-to-fetus *in-utero* transmission of CMV and clinical significance of IgG avidity testing in diagnosis of congenital CMV infections.

## 2. Epidemiology of Maternal and Fetal CMV Infection

Due to the fact that CMV is less contagious, transmission occurs via close contact with viral contaminated human secretions and bodily fluids. The virus can be found in urine, saliva, blood, cervical secretions, semen, breast milk and transplanted tissues/organs. These secretions and bodily fluids intermittently contain or excrete CMV [4,5]. CMV excretion is usually prolonged after acute primary infection but this rarely occurs with recurrent or secondary infections [4,5]. Transmission of CMV to fetuses and neonates occurs in one of following modes (1) *in-utero* through placenta from blood spread to the fetus during maternal viremia; (2) at birth by during delivery after exposure to infected cervical and vaginal secretions; (3) postnatally by ingestion of CMV-positive saliva via kissing or breast milk and even transfusion of infected blood. These can persist for up to 18 months of age [6,7].

CMV infection has worldwide endemic distribution and lacks seasonal variation [8]. In developed societies, around 50%–60% of pregnant, middle to high class women have developed antibodies to CMV, compared with over 70% of those from lower socioeconomic categories [9]. Babayo *et al*. demonstrated that 2.2% and 79.1% of pregnant women had anti-CMV IgM and IgG antibodies, respectively at Maiduguri, Nigeria [10]. Overall, it has been estimated that 1%–4% of IgG seronegative women are affected with primary CMV infection during pregnancy, and around 30%–40% of the fetus are transplacentally infected. Out of this, about 10% infants manifest clinical symptoms at birth [9,10]. Approximately 1%–3% of infants born of women with preexisting antibody (IgG seropositive) to CMV are infected *in-utero*, but they are rarely symptomatic at birth (<1%) [8]. It has been known that the risk of symptomatic CMV infections at birth or the children who will develop sequelae are higher if maternal infection occurs during the first eight weeks of pregnancy, and also most of severe congenital CMV infections are caused by primary rather than secondary infection of pregnant women [4,10].

## 3. Stages of CMV Infections

### 3.1. Primary CMV Infection

Primary CMV infection occurs when recent/ acute infection occurs in an individual previously confirmed to be anti-CMV IgG negative by ELISA. The appearance of specific anti-CMV IgM antibodies in a seronegative person is presumptive diagnosis of primary CMV provided that passive transfer of antibodies via immunoglobulin or blood products can be excluded [11]. Due to significant false positivity encountered in CMV IgM assays, a negative IgM excludes recent infection but a positive IgM requires IgG avidity testing to confirm a primary infection.

### 3.2. Recurrent Infection

CMV infection is said to be recurrent when serological or molecular tests detect new CMV infection in an individual who had previous documented anti-CMV IgG antibodies at least 4 weeks during active surveillance. Recurrent infection may result from reactivation of latent CMV (endogenous) or reinfection (exogenous) [11].

### 3.3. Reinfection

“Reinfection” is defined as detection of a new CMV strain that is distinct from that which caused the patient’s original infection. CMV reinfection can be determined using molecular sequencing of specific regions of the viral genome or by detecting polymorphic genes of the viral infection. It is diagnosed when the 2 CMV strains are genetically distinct [11].

### 3.4. Reactivation

Reactivation is assumed when an individual with history of IgG seropositivity develops active infection from strains that are found to be indistinguishable either by sequencing specific regions of the viral genome or by using a variety of molecular techniques that examine genes known to be polymorphic. Reactivated infections are said to be due to latent CMV from patients’ nervous ganglion [11].

## 4. Clinical Manifestations of Congenital Cytomegalovirus (CCMV) Infection

It has been estimated that about 60%–90% of children with symptomatic and 10%–15% asymptomatic CCMV infection in the neonatal period will develop long-term neurological damage when followed up at birth [12]. Sensorineural hearing loss (SNHL), seizures, chorioretinitis, mental retardation, speech and psychomotor delays, learning disabilities and dentition disorders are the most frequent long-term consequences of CCMV [12]. SNHL is the most common CMV consequence that does not manifest at neonatal stage but in many cases may fluctuate and be progressive in nature, becoming clinically apparent in later childhood (during the first six years of life) [13].

The overall prevalence of both symptomatic and asymptomatic SNHL caused by CCMV infection at birth is 5.2% and late-onset hearing loss at six years was found to be 15.4% [14]. Generally, children with symptomatic CMV infection experience auditory loss at earlier age and with greater severity than those with asymptomatic infection [9]. Previous cohort studies showed that 40%–58% of neonates with symptomatic Congenital CMV infection will suffer from severe neurological consequences and 5%–30% mortality rates [15].

It is also now recognized that asymptomatic CCMV infection is associated with increased risk of SNHL [16]. In particular, different studies report that 6%–25% of asymptomatic children will develop late-onset sequelae. The neurological sequelae (particularly SNHL) place CCMV infections the major non-genetic and viral etiology of neurological disorders in children [14,15,16,17,18,19,20].

Little attention has been given to the influence of CCMV infection on children’s physical growth and intellectual development. Shan *et al.* investigated changes in audiology, nervous behavior, intellectual development, and behavioral development in order to find out the impacts of asymptomatic CCMV infection [15]. In an analysis of 180 children with symptomatic CCMV infection, 48% were noted to have hearing defects on follow-up. Of these children, 30% (*i.e.*, 26 of 87) had delayed-onset hearing loss while the remaining 61 (70%) had hearing loss at birth or in the newborn period. Progressive hearing loss was observed in 63% (55 of 87) children [21].

Four other studies demonstrated that a high viral load in early infancy in form of high CMV viruria (450,000 PFU/mL) is largely predictive of audiologic disorders [14,21,22,23]. Greater than 70% of symptomatic (with or without CNS involvement) infants with viruria of >5 × 10^4^ PFU⁄mL will have poor neurodevelopmental outcome when compared with only 4% with viruria of <3.5 × 10^3^ PFU⁄mL [22,24].

## 5. Laboratory Investigations of CCMV Infections

The diagnosis of CCMV infection in children is based on the ability to demonstrate CMV activities by cell culture isolation from urine specimens, molecular detection of CMV-DNA using PCR in bodily fluids sampled before 3 weeks of age or by detection of structural antigens or anti-CMV specific IgM in blood [25]. However, since 2010 the gold standard diagnostic test for CCMV is PCR on saliva or urine. Anti-CMV IgM test alone is no longer useful to diagnose CCMV infection. Because PCR is too sensitive and may give false positive results, it has been suggested that PCR testing of urine of newborns for CMV should only be used alongside viral culture.

A rapid diagnosis with low sensitivity may be obtained by detection of CMV antibody in blood of neonates. IgG antibodies are mostly maternally transferred antibodies, while the demonstration of IgM antibodies in the newborn is indicative of congenital infection, because maternal IgM antibodies can’t be transplacentally acquired. However, only 70% of neonates have detectable anti-CMV IgM antibodies at birth [26]. With regards to the mother, seroconversion of CMV IgG between two sera samples obtained 2–3 weeks apart provides the most reliable diagnosis of primary CMV infection. The presence of anti-CMV-IgM antibodies is suggestive of acute, recent or ongoing infection, but they have questionable specificity and sensitivity particularly in cases of false positive IgM results that arise from cross reaction with Epstein-Barr virus (EBV) and Herpes Simplex Virus (HSV). These limitations restrict the usefulness of CMV IgM tests and invariably warrant the need for further confirmative diagnosis of maternal primary CMV [26].

The CMV-IgG avidity test has also been successfully used to diagnose CCMV in children. Vilibic-Cavlek *et al* evaluated the value of IgG avidity in diagnosis of CCMV infection in newborns and infants [27]. They collected and analyzed serum samples from 40 under 1 year old infants with suspected CCMV infection. 13 (32.5%) of the subjects were seropositive to anti-CMV IgM, 3 (7.5%) had equivocal IgM antibodies result, and 24 (60.0%) patients had IgG antibodies only. Upon using IgG avidity testing, CMV infections (with low avidity indices) were documented in 61.5% IgM positive and 54.2% IgM negative infants [27]. IgG avidity distribution across age of infants demonstrated recent primary CMV infection in 58.8% patients younger than 3 months compared with 91.7% and 81.8% in 3–6 and 6–12 months old infants respectively. The study showed that IgG avidity testing of children older than 3 months of age was significantly useful for CMV diagnosis regardless of their IgM result. However, in infants <3 months of age, transplacentally derived maternal IgG antibodies with high avidity index influenced IgG avidity result of the children. They concluded that CMV diagnosis should be confirmed by further virological tests such as cell culture isolation or molecular assay [27]. For children >3 weeks of age, the CMV-IgG test may be useful to diagnose a CMV infection but cannot precisely determine whether the infection is congenital or postnatal.

## 6. Clinical and Laboratory Findings in Maternal CMV Infection

Most maternal CMV infections are asymptomatic even during the acute stage. Less than 5% of pregnant women with primary CMV are reported to be symptomatic, and fewer suffer from mononucleosis-like illnesses [28]. Most common symptoms include persistent fever, myalgia, cervical lymphadenopathy and malaise, and, less commonly, hepatitis and pneumonia have been implicated [29]. Hematological and biochemical testing may sometimes disclose atypical lymphocytosis and slightly raised alanine and aspartate amino transferases activities. Virological and serological tests are the best means of establishing diagnosis [29].

Primary CMV infection is likely to occur when there is seroconversion from IgM negativity to IgM positivity in combination with low IgG avidity index [30,31,32]. The anti-cytomegalovirus IgG avidity test is presently the most reliable investigation used to identify primary CMV infection in pregnant women [32,33]. The IgG avidity test is highly specific (100%) and sensitive (94.3%). The degree of antibody avidity slowly and progressively increases reflecting the maturation of humoral immune response over time. Low avidity indices indicate low maturation of IgG antibodies in blood caused by recent or acute primary CMV infection (Table 1) [30,33]. Low avidity indices are encountered 18–20 weeks after the onset of symptoms in apparently healthy individuals [33].

The determination of anti-CMV IgG avidity performed before the 16–18th week of pregnancy identifies all women who will have primary maternal infection with sensitivity of 100%. After 20 weeks’ gestation, sensitivity drastically reduces to around 62.5% [34]. A high IgG avidity index between the 12th and 16th week of gestation is considered a good indicator of past infection [35].

Virological testing plays a secondary role in establishing primary maternal CMV infection. CMV isolation using cell culture of urine or cervical secretions is a poor indicator of the risk of transplacental transmission and severe fetal damage. [36] CMV detection in blood specimen by viral isolation and search for viral components by the antigenaemia tests and molecular assays failed to correlate with the clinical course of infection, risk of *in-utero* transmission and severity of fetal/neonatal complications [37]. Both antigenaemia and PCR tests had low sensitivity of 14.3% and 47.6%, respectively, for detecting congenital CMV transmission in a group of pregnant women who contracted primary CMV infection between the 4th and 30th week of gestation. Positive and negative prediction rates were also poor [31]. These findings suggested that maternal primary CMV may or may not be detected at the time of diagnosis. Positive viral detection was not associated with higher risk of infection and fetal/neonatal complications [31].

Screening for CMV by serology has been a controversial issue. Globally, routine serological testing of pregnant women has never been recommended by most public health authorities [37,38]. If this screening is to be considered, it should be performed at the early stage of pregnancy or even prior to a planned pregnancy. If a woman is seronegative, repeated examinations during pregnancy should be done when there is clinical suspicion. However, screening is usually done before pregnancy for diseases such as rubella and varicella against which immunization can be provided, whereas there is currently no effective and safe vaccine for CMV [38]. Moreover, because effective prenatal treatment options are not yet available, the choices when a woman is carrying a baby with CMV infection or disease are limited to elective termination of the pregnancy or expectant observation until delivery. Prenatal testing, however, offers an opportunity to educate women about behavioral and precautionary suggestions for seronegative women [39]. Routine antibody testing, especially if done before pregnancy, helps to differentiate primary from non-primary infection in pregnant women suspected of CMV [40]. Naessens *et al.* evaluated a screening program for CMV in which serological testing was performed at the first prenatal visit; findings from their studies showed that such a program allowed detection of 82% of all congenital acquired CMV infections [41].

## 7. The IgG Avidity Test

The functional binding affinity of anti-CMV IgG antibodies increases progressively over time after immunity by infection; it is otherwise referred to as maturation of the humoral immune response [42]. Low IgG antibodies avidity indices may indicate primary infection whereas high avidity indices indicate non-primary infection. In several previous evaluations, avidity indices (AI) above 60% during the first trimester of pregnancy could reasonably be considered a good indicator of past CMV infection, whereas in women with low AI less than or equal to 50%, there was a risk of congenital CMV transmission. [40,43,44,45]. There is frequent clinical use of CMV avidity tests in most developed nations like Israel, France, Germany, United States of America (US) and the Netherlands. For instance, in the US, clinical CMV avidity testing is readily available from Focus Diagnostics (Cypress, CA, USA). CMV IgG avidity test is an FDA Class II diagnostic test. Among specialists in human genetics, infectious disease and obstetrics involved in the diagnosis and treatment of CCMV infections, there is a general knowledge of IgG avidity tests and how to use test results to counsel patients. However, there paucity of information (particularly in underdeveloped and developing countries of Africa) in regards to the use of CMV IgG avidity tests in the investigations of maternal and congenital CMV infections.

### 7.1. Quality Control Measures in IgG Avidity Testing

The recommended control requirement for the CMV IgG avidity assay is that a single sample of each control be tested once every 24 h each day of use. Controls are ordered as multiconstituent controls for “CMV Avidity”.

### 7.2. Interpretation of Results

Avidity index to anti-CMV IgG test result are mainly interpreted as follows, even though slight adjustment of cut-off values vary and some manufacturers changed these values due to extensive and independent clinical validations.

(a) Less than 50.0% Avidity: Low avidity
(b) 50.0%–59.9% Avidity: Gray zone
(c) Greater or equal to 60.0% Avidity: High avidity.

## 8. Findings of CMV IgG Avidity Testing from Previous Studies

This was done using an extensive internet search of relevant published articles through PubMed, Scopus and HINARI on the concepts of mother-to-fetus *in-utero* transmission of CMV and clinical significance of IgG avidity testing in diagnosis of maternal and congenital CMV infections (Table 2).

## 9. Conclusions

Congenital CMV infection is a leading infectious etiology of mental retardation and sensorineural deafness in children. It has been established that primary CMV infections have the highest propensity for congenital transmission and are more likely to cause fetal complications than recurrent or reactivated infections. Positive anti-CMV IgM and low IgG avidity indices less than 50% are diagnostic of primary maternal CMV infection. There is paucity of information in regards to the use of anti-CMV IgG avidity tests in developing countries, consequently limiting the serodiagnostic power of maternal primary CMV infections leading to inadequacy in assessing risk for CCMV transmission. Once primary CMV infection with high viral load has been established, congenital fetal infection is most likely to occur. These findings will help clinicians to counsel pregnant women infected with CMV about the likely outcome of their fetuses and enable couples themselves to decide the future of the pregnancy.

## 10. Recommendations

The quality of findings we reported in this review can lead to the following recommendations:

1. Behavioral interventions and hygienic practices should be advocated to pregnant women for effective prevention of CMV infections.

2. Clinical use of serological tests should be in pregnant women suspected of CMV infection especially those with mononucleosis-like symptoms or sonographic findings suggestive of CMV infection. Serodiagnosis of primary maternal CMV infection should be based on the detection of anti-CMV IgM antibody coupled with low IgG avidity index while diagnosis of congenital CMV should be based on detection of CMV DNA in saliva or urine of neonates or serum IgM positivity/low IgG avidity index.

## Figures and Tables

**Table 1:** Interpretation of CMV serological test results.

S/No	CMV IgG	CMV IgM	CMV IgG Avidity	Indication for
1	Nonreactive	Nonreactive	N/A	No infection
2	Reactive	Nonreactive	High avidity	Past infection; low risk for *in-utero* transmission
3	Reactive	Reactive	Low avidity	Primary infection; high risk for *in-utero* transmission
4	Reactive	Reactive	High avidity	non-primary infection; low risk for *in-utero* transmission

Source: Guideline for serological diagnosis of congenital CMV infections, Centers for Diseases Control and Prevention, 2010 [25].

**Table 2:** Diagnostic values of anti-CMV IgG avidity testing from previous studies.

S/No.	Study Design	No. of Subjects	Percentage Anti-CMV IgG+	Percentage Anti-CMV IgM+	Percentage IgM+ with Low IgG Avidity Indices	Percentage IgM+ with High IgG Avidity Indices	Inference	Reference
1	Cohort study	6067	58%	3.0%	2.0%	1.0%	Anti-CMV IgM and IgG avidity testing was useful for evaluating primary CMV infection.	Dollard *et al.* [46]
2	Cross-sectional study	879	17.6%	0.91%	0.68%	0.23%	Pregnant women had non-primary CMV infection (*i.e.*, IgG+ and IgM+)	Ozekinci *et al.* [47]
3	Case-control study	43 women with RPL and 43 aged match control participants with no history of abortion	90.6% *vs.* 69.8% for case *vs.* control respectively	2.3% each from both group	None from both group	95.3% *vs.* 93.0%	No association was found between IgG Avidity Indices and RPL	Sherkat *et al.* [48]
4	Cohort study	600	56.8%	5.5%	1.2%	4.3%	Established primary CMV infection and high risk of congenital transmission	Munro *et al.* [35]
5	Cross-sectional	2817	68.3%	0.6%	0.46%	0.14%	IgG avidity testing crucial for assessing risk for congenital transmission	*Paschale et al.* [49]
6	Cross-sectional	546	100%	7.3%	none	7.3%	IgM+ subjects had no Primary infection as evidenced by high IgG avidity indices	Kamel *et al.* [50]
7	Cohort	744	98.1%	1.7%	none	1.7%	Maternal primary CMV infection was not detected. CMV IgG avidity test enabled the identification of women with low risk of congenital transmission	Seo *et al.* [51]
8	Case-control study	527 pregnant women with adverse pregnancy/neonatal outcomes and 496 mothers of healthy infants	99.4% *vs.* 98.0%	3.8% *vs.* 1.6%	0.9% *vs.* 0.0%	2.1% *vs.* 1.6%	IgM positivity were recurrent infections (*i.e.* IgG+ and IgM+)	Zhang *et al.* [52]

RPL = Recurrent pregnancy loss; *vs.* = *versus*; + = Seropositivity; IgM = Immunoglobulin M; IgG = Immunoglobulin G.
